# Supplementary material for: Core control principles of the eukaryotic cell cycle
Source: Nature. 2022 Jun 8;607(7918):381–6. doi: 10.1038/s41586-022-04798-8 (PMC9279155; doi:10.1038/s41586-022-04798-8)
Supplement: Supplementary file 1 — This file contains Supplementary Fig. 1 (unprocessed and uncropped blot scans, relating to Extended Data Fig. 1) and the full legends for Supplementary Tables 1–3. [file 41586_2022_4798_MOESM1_ESM.pdf]

nature portfolio
https://doi.org/10.1038/s41586-022-04798-8

Supplementary information

Core control principles of the eukaryotic cell cycle

In the format provided by the authors and unedited

Supplementary information for:

Core Control Principles of the Eukaryotic Cell Cycle

Souradeep Basu[1,3*], Jessica Greenwood[1], Andrew W. Jones[1], and Paul Nurse[1,2]

1 Cell Cycle Laboratory, The Francis Crick Institute, 1 Midland Road, London, NW1 1AT, UK.

2 Laboratory of Yeast Genetics and Cell Biology, Rockefeller University, 1230 York Ave, New York, NY 10065, USA.

3 Present address: DeepMind, London, UK

*Correspondence to souradeepb@deepmind.com

Supplementary Figures

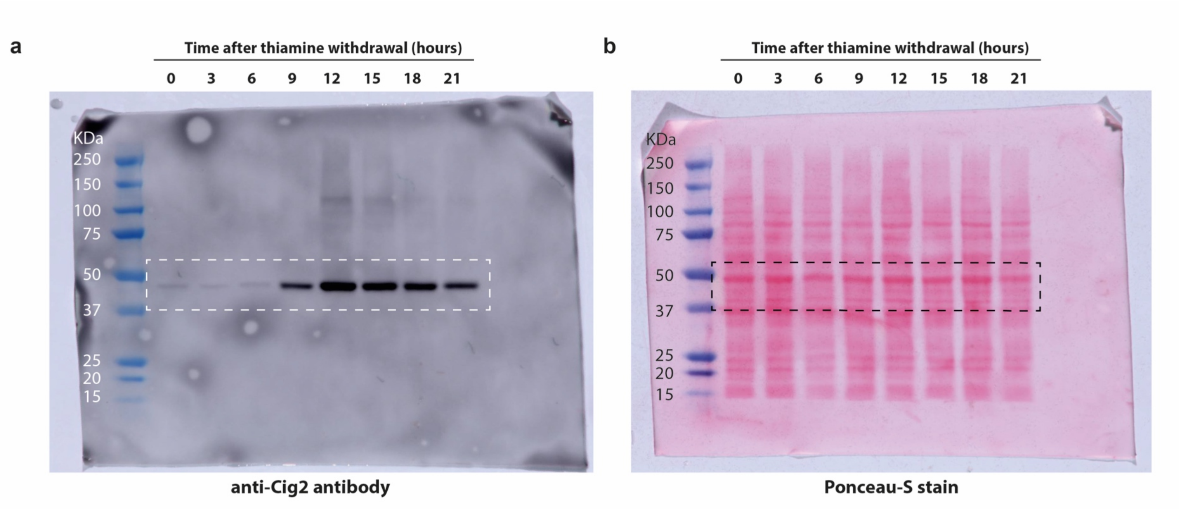

Supplementary Figure 1 – Unprocessed and uncropped blot scans, relating to Extended Data Figure 1

a) Western blot scan for Extended data, Figure 1b, upper panel. Dashed box gives area used in original figure. Molecular weight markers given on scan.

b) Ponceau-S blot scan for Extended data, Figure 1b, lower panel. Dashed box gives area used in original figure. Molecular weight markers given on scan.

Supplementary Table Legends

Supplementary Table 1 –CDK site classification upon S-CDK and M-CDK induction

Sheet 1: Overview of CDK site clustering and S-CDK or M-CDK preference data.

Sheet 2: Complete dataset used in Figure 2. Entire dataset without any data omissions was used for clustering. Up to one outlier data point per trace was omitted for representation in Figure 2b-d.

Supplementary Table 2 – Influence of Cut12$^{\Delta PP1}$ on hypophosphorylated S-CDK sites

Lists 35 phosphosites that were identified as hypophosphorylated by S-CDK, and the influence of centrosomal PP1 removal on their phosphorylation.

Supplementary Table 3 – *S. pombe* strains and expression plasmids

Sheet 1*: S. pombe* strains used in this study, and their genotype.

Sheet 2: Plasmids used for tetracycline-induced expression of cyclin-CDK constructs.
