# Supplementary material for: Core control principles of the eukaryotic cell cycle
Source: Nature. 2022 Jun 8;607(7918):381–6. doi: 10.1038/s41586-022-04798-8 (PMC9279155; doi:10.1038/s41586-022-04798-8)
Supplement: Supplementary file 2 — Reporting Summary [file 41586_2022_4798_MOESM2_ESM.pdf]

nature portfolio

Corresponding author(s): Souradeep Basu

Last updated by author(s): Mar 26, 2022

Reporting Summary

Nature Portfolio wishes to improve the reproducibility of the work that we publish. This form provides structure for consistency and transparency in reporting. For further information on Nature Portfolio policies, see our Editorial Policies and the Editorial Policy Checklist.

Statistics

For all statistical analyses, confirm that the following items are present in the figure legend, table legend, main text, or Methods section.

n/a	Confirmed	
☐	☒	The exact sample size (n) for each experimental group/condition, given as a discrete number and unit of measurement
☐	☒	A statement on whether measurements were taken from distinct samples or whether the same sample was measured repeatedly
☒	☐	The statistical test(s) used AND whether they are one- or two-sided<br>*Only common tests should be described solely by name; describe more complex techniques in the Methods section.*
☒	☐	A description of all covariates tested
☐	☒	A description of any assumptions or corrections, such as tests of normality and adjustment for multiple comparisons
☐	☒	A full description of the statistical parameters including central tendency (e.g. means) or other basic estimates (e.g. regression coefficient) AND variation (e.g. standard deviation) or associated estimates of uncertainty (e.g. confidence intervals)
☒	☐	For null hypothesis testing, the test statistic (e.g. F, t, r) with confidence intervals, effect sizes, degrees of freedom and P value noted<br>*Give P values as exact values whenever suitable.*
☒	☐	For Bayesian analysis, information on the choice of priors and Markov chain Monte Carlo settings
☒	☐	For hierarchical and complex designs, identification of the appropriate level for tests and full reporting of outcomes
☒	☐	Estimates of effect sizes (e.g. Cohen's d, Pearson's r), indicating how they were calculated

Our web collection on statistics for biologists contains articles on many of the points above.

Software and code

Policy information about availability of computer code

Data collection	Microscopy images were collected using ImageJ 1.52j (NIH). MaxQuant (version 1.6.14.0) was used for mass spectrometry data processing.
Data analysis	Microscopy data was analysed using ImageJ 1.52j (NIH). Mass spectrometry data was analysed with Perseus (version 1.6.14.0). GraphPad Prism (Versions 7 and 8) were used for data representation.

For manuscripts utilizing custom algorithms or software that are central to the research but not yet described in published literature, software must be made available to editors and reviewers. We strongly encourage code deposition in a community repository (e.g. GitHub). See the Nature Portfolio guidelines for submitting code & software for further information.

Data

Policy information about availability of data

All manuscripts must include a data availability statement. This statement should provide the following information, where applicable:

- Accession codes, unique identifiers, or web links for publicly available datasets
- A description of any restrictions on data availability
- For clinical datasets or third party data, please ensure that the statement adheres to our policy

All mass spectrometry proteomics data generated have been deposited to the ProteomeXchange Consortium via the PRIDE partner repository with the dataset identifier PXD029073.

Field-specific reporting

Please select the one below that is the best fit for your research. If you are not sure, read the appropriate sections before making your selection.

☒ Life sciences ☐ Behavioural & social sciences ☐ Ecological, evolutionary & environmental sciences

For a reference copy of the document with all sections, see nature.com/documents/nr-reporting-summary-flat.pdf

Life sciences study design

All studies must disclose on these points even when the disclosure is negative.

Sample size	Sample sizes were not predetermined, however we aimed to count 100 cells per time-point to give an accurate reflection of cell population behaviour.
Data exclusions	Known CDK sites were excluded from the phosphoproteomic analysis if they: a) displayed consistent aberrant phosphorylation behaviour upon Cyclin-CDK induction, or b) displayed negative phosphorylation values. This led to ~9% data loss.
Replication	All attempts at replication were successful, and all experiments were biologically replicated at least twice.
Randomization	Randomisation was not relevant to this study, as single genotypes was under study in any given experiment. Therefore, each group only contained a single member.
Blinding	Blinding was not possible in this study, as the phenotypes displayed are obvious indicators of the genotype under study.

Reporting for specific materials, systems and methods

We require information from authors about some types of materials, experimental systems and methods used in many studies. Here, indicate whether each material, system or method listed is relevant to your study. If you are not sure if a list item applies to your research, read the appropriate section before selecting a response.

Materials & experimental systems

n/a	Involved in the study	
☐	☒	Antibodies
☐	☒	Eukaryotic cell lines
☒	☐	Palaeontology and archaeology
☒	☐	Animals and other organisms
☒	☐	Human research participants
☒	☐	Clinical data
☒	☐	Dual use research of concern

Methods

n/a	Involved in the study	
☒	☐	ChIP-seq
☒	☐	Flow cytometry
☒	☐	MRI-based neuroimaging

Antibodies

Antibodies used	α-Cig2 (mouse monoclonal, primary Ab) (abcam, CIG 3A11/5, Cat# ab10881)<br>α-mouse IgG (goat polyclonal, secondary Ab) (AbD SeroTEC, Cat# STAR120)
Validation	Antibody was validated by manufacturer (https://www.abcam.com/cig2-antibody-cig-3a115-ab10881.html)

Eukaryotic cell lines

Policy information about cell lines

Cell line source(s)	All S. pombe strains used are outlined in Supplementary Table 3. The L972 fission yeast strain background was used in all experiments
Authentication	All strains were authenticated by PCR, by positive antibiotic selection, and by negative auxotrophic selection.
Mycoplasma contamination	S. pombe cultures do not get contaminated by mycoplasma.
Commonly misidentified lines<br>(See ICLAC register)	None.
